# A new approach to modeling transdermal ethanol kinetics

**DOI:** 10.14814/phy2.70070

**Published:** 2024-10-02

**Authors:** Joseph C. Anderson

**Affiliations:** ^1^ Department of Bioengineering University of Washington Seattle Washington USA

**Keywords:** blood alcohol concentration, diffusion, fuel cell, mathematical modeling, skin alcohol

## Abstract

Measurement of ethanol above the skin surface (supradermal) is used to monitor blood alcohol concentrations (BAC) in both legal and consumer settings. Previously, the relationship between supradermal alcohol concentration (SAC) and BAC was described using partial and ordinary differential equations (PDE model: J. Appl. Physiol. 100: 649‐55, 2006). Using a range of BAC profiles by varying absorption times and peak concentrations, the PDE model accurately predicted experimental measures of SAC. Recently, other mathematical models have relied on the PDE model. This paper proposes a new approach to modeling transdermal ethanol kinetics using a mass transfer coefficient and only ordinary differential equations (ODE model). Using a range of BAC profiles, the ODE model performed very similarly to the PDE model. The ODE model had slightly slower washout rates and slightly slower times to peak SAC and to zero SAC. Similar to the PDE model, a sensitivity analysis on the ODE model showed changes in solubility and diffusivity within the stratum corneum, stratum corneum thickness, and the volume of gas above the skin affected model performance. This new model will streamline integration into larger physiologic models, reduce computation time, and decrease the time to transform skin alcohol measurements to blood alcohol concentrations.

## 1 INTRODUCTION

Alcohol content in the body is typically measured using invasive blood or less invasive breath samples. While well‐controlled samples can produce accurate measures of alcohol content (Hlastala & Anderson, 2016), these measures only provide a snapshot in time. In addition to appearing in blood and breath, alcohol can appear above the skin surface by diffusion from subcutaneous capillaries through the skin tissue and into the supradermal air. Because alcohol continually diffuses across the skin surface, measurement of alcohol above the skin can provide a semi‐continuous, non‐invasive method for monitoring blood alcohol content. In legal and medical settings, devices measuring supradermal alcohol concentration (SAC) are used to evaluate abstinence from alcohol consumption (Dougherty et al., 2014; Marques & McKnight, 2007). Consumer products allow users to monitor their alcohol concentration during and after consumption (Wang et al., 2019). For these measurements to predict alcohol consumption or blood alcohol concentration (BAC), the relationship between SAC and BAC must be understood.

A previously developed mathematical model (i.e., PDE model) demonstrated that diffusion was the primary mechanism of alcohol movement across the skin. The PDE model described diffusion of alcohol from the blood, across the skin tissue and into the supradermal air using a mix of ordinary differential (ODE) and partial differential equations (PDE) (Anderson & Hlastala, 2006). The PDE model was validated by demonstrating close agreement between model predictions and experimental data from the scientific literature. Both the model and experimental data showed that the peak SAC was attenuated and delayed relative to BAC. Additionally, the decrease in SAC was slower than the metabolic elimination rate and the time to return to a zero concentration was delayed as compared to BAC.

Since publication, the PDE model has been utilized for a variety of applications. Scientifically, the PDE model explained the physiological relationship between SAC and BAC. The effects of anatomical and physio‐chemical parameters on alcohol movement across the skin were used to explain the delays and attenuation of SAC relative to BAC (Karns‐Wright et al., 2017; Marques & McKnight, 2009). Using measurements of SAC over time (i.e., SAC profile), a deconvolution algorithm was developed based off the PDE model to predict profiles of breath alcohol concentration (BrAC) (Dumett et al., 2008). While algorithms deconvolving SAC to BrAC may or may not (i.e., blind deconvolution) use training datasets, the mathematics are computationally intensive (Dumett et al., 2008; Rosen et al., 2014). In addition to deconvolution, investigators, studying whole body alcohol kinetics, incorporated the PDE model into a larger model composed of ODEs that describe alcohol movement within the body. These studies evaluated the effects of absorption, metabolism, sex, and body mass on the appearance of alcohol on the skin surface (Webster & Gabler, 2007, 2008).

While partial differential equations provide the most accurate description of alcohol diffusion across the skin, the PDE model inhibits economical and efficient integration in low‐power settings (e.g., mobile devices) or within larger models that only use ODEs. Solving systems of PDEs via numerical integration requires simultaneous solution of hundreds of equations which is computationally intensive and inefficient for implementation on a low‐powered alcohol bracelet. Likewise, integrating the PDE model into a system of ODEs for whole body alcohol kinetics can be cumbersome. The mismatch in spatial resolution creates an impedance to data flow between models. Assumptions must be made so that the output from the low spatial resolution (i.e., compartmental) ODE model can be used as input into the high spatial resolution PDE model and vice versa. In addition to data flow, the differences in spatial gradient descriptions between the two models means different numerical algorithms are required to solve for the underlying alcohol concentrations (Anderson et al., 2003; Carlson et al., 2008).

The new approach described each skin compartment using a single ODE and alcohol concentration. The key to this approach was defining a mass transfer coefficient to describe diffusion between skin compartments. The performance of the ODE model was compared to that of the PDE model using a range of blood alcohol profiles where absorption times and peak concentrations were varied. Additionally, a sensitivity analysis using Latin hypercube sampling was performed on the ODE model and the results were compared to a similar analysis performed on the PDE model. Differences in model performance and factors affecting prediction of BAC from SAC were discussed.

## 2 METHODS

### 2.1 Mathematical model

**FIGURE 1:**
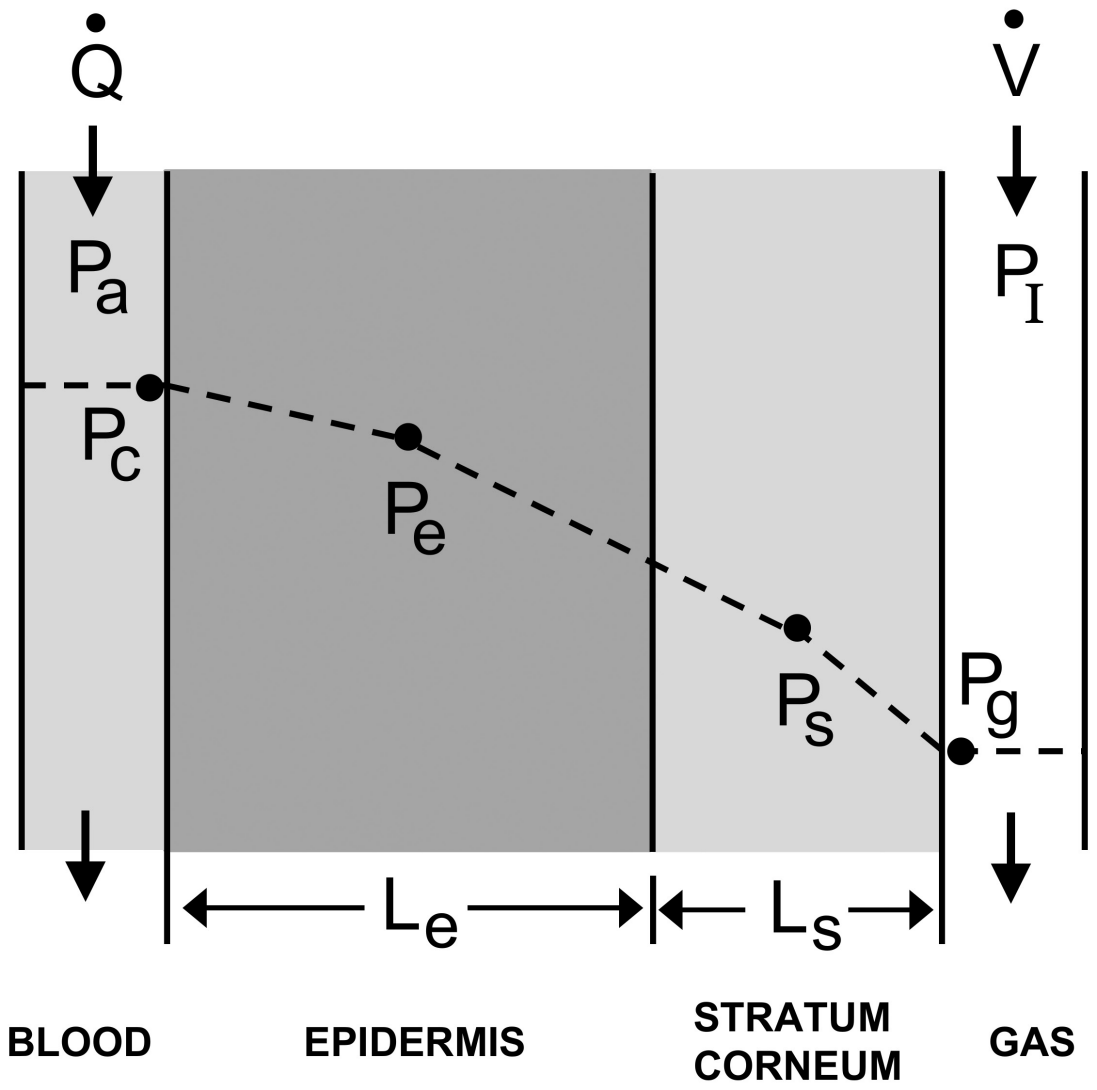
Schematic of ethanol transport from blood, through two skin layers, and into the supradermal airspace. Blood flow ($\dot{Q}$) delivers ethanol at partial pressure *P*_a_ into the capillary where ethanol diffuses through the epidermis and stratum corneum before entering the gas compartment. Fresh gas (*P*_I_ = 0) flowing at a rate $\dot{V}$ above the skin removes ethanol from the system. Ethanol partial pressures in the capillary (*P*_c_) and gas compartment (*P*_g_) are well mixed. Ethanol partial pressures in the epidermis (*P*_e_) and stratum corneum (*P*_s_) are the partial pressures at the center of the compartment. The linear concentration gradient between epidermis and stratum corneum is an assumption required for the mass transfer coefficient. See Table 1 for more definitions.

The skin model is composed of four compartments: blood, epidermis, stratum corneum, and gas (Figure 1). Blood flow through the capillary delivers dissolved ethanol to the epidermis. Ethanol flows through the epidermis and the stratum corneum via diffusion before it reaches the gas compartment, which is ventilated with fresh air. Ethanol transport from sensible perspiration (i.e., sweating) is ignored. Capillary and gas compartments were assumed to be individually well‐mixed. Ethanol partial pressures in the epidermis (*P*_e_) and stratum corneum (*P*_s_) are the partial pressures at the center of the compartment.

For this new approach, the mathematical model of ethanol kinetics across the skin was created by writing four ODEs to describe conservation of mass within each layer. To allow mass transfer between skin compartments, a mass transfer coefficient between the epidermis and stratum corneum was defined (below). The prior PDE model consisted of a mixture two PDEs and two ODEs (Anderson & Hlastala, 2006). For that model, the spatial derivatives and boundary conditions eliminated the need for a mass transfer coefficient.

A mass transfer coefficient was derived using Fick's first law of diffusion (Bird et al., 1960; Bui et al., 1998). Assuming a linear concentration profile between compartments, the flux of mass was written as the molecular diffusion coefficient divided by the diffusion length and multiplied by the partial pressure difference across the diffusion length. The molecular diffusivity, D_e_ for epidermis and D_s_ for stratum corneum, divided by the thickness of the layer, *L*_e_ for epidermis and *L*_s_ for stratum corneum, can be thought of as a conductance that is equivalent to the mass transfer coefficient. The solubility of ethanol in each compartment is described by *β*_e_ and *β*_s_ for the epidermis and stratum corneum, respectively. Thus, the transfer coefficient is composed of two conductances; one corresponding to half of the epidermis layer and another corresponding to half of the stratum corneum layer. Because conductances add as their inverse, the mass transfer coefficient between the epidermis and stratum corneum, *k*_e,s_, can be written as follows:

$k_{e,s}={\left[\frac{L_{e}}{2D_{e}\beta_{e}}+\frac{L_{s}}{2D_{s}\beta_{s}}\right]}^{-1}.$

Four coupled ODEs represent the movement of ethanol between blood, epidermis, stratum corneum, and gas. Figure 1 schematically describes this model, in which ethanol dissolved in blood is delivered to the capillary compartment at a flow rate $\dot{Q}$ (mL/s) and ethanol appears via diffusion into the gas compartment from which gas is removed at a rate $\dot{V}$ (mL/s). Equation (1) describes the rate of change of mass (*β*·*V*·*P*) of ethanol in the capillary blood compartment. It is equal to the rate of ethanol perfusing the capillary space via blood flow and the rate of ethanol leaving the blood compartment from blood flow and diffusion across the capillary membrane into the epidermis. The partial pressure of ethanol entering and leaving the blood compartment is *P*_a_ and *P*_c_, respectively. The solubility of alcohol in blood is *β*_b_. The blood compartment has a thickness equal to the diameter of a blood cell (*L*_c_ = 0.0007 cm) and a surface area of A_c_.

(1) $$\beta_{b}\;A_{c}L_{c}\frac{{\mathrm{dP}}_{c}}{\mathrm{dt}}=\overset{•}{Q}\;\beta_{b}\;\left(P_{a}-P_{c}\right)-\frac{D_{e}\beta_{e}A_{c}}{\frac{1}{2}L_{e}}\left(P_{c}-P_{e}\right)$$

Equations (2) and (3) describe accumulation of ethanol in the epidermis and stratum corneum, respectively, via diffusion of ethanol into and out of each compartment. The partial pressures of alcohol in each compartment are *P*_e_ for the epidermis and *P*_s_ for the stratum corneum. The tissue and gas compartments have the same surface area (*A* = 1 cm^2^).

(2) $$\beta_{e}{\mathrm{AL}}_{e}\frac{{\mathrm{dP}}_{e}}{\mathrm{dt}}=\frac{D_{e}\beta_{e}A_{c}}{\frac{1}{2}L_{e}}\left(P_{c}-P_{e}\right)‐k_{e,s}A\left(P_{e}-P_{s}\right)$$

(3) $$\beta_{s}{\mathrm{AL}}_{s}\frac{{\mathrm{dP}}_{s}}{\mathrm{dt}}=k_{e,s}A\left(P_{e}-P_{s}\right)-\frac{D_{s}\beta_{s}A}{\frac{1}{2}L_{s}}\left(P_{s}-P_{g}\right)$$

Equation (4) describes accumulation of ethanol in the air space above the skin and enclosed by the ethanol measurement device. The rate of change of ethanol in this compartment is determined by addition of ethanol from the ambient air (*P*_I_ = 0), subtraction of ethanol removed by a pump for analytical measurement, and addition of ethanol diffusing across the air‐skin interface from the stratum corneum, adjacent to the compartment. *P*_g_ is the partial pressure of gas, *L*_g_ is the thickness of the compartment and *β*_g_ is the solubility of ethanol in gas. Table 1 provides parameters definitions and their associated units.

(4) $$\beta_{g}\;{\mathrm{AL}}_{g}\frac{{\mathrm{dP}}_{g}}{\mathrm{dt}}=\overset{•}{V}\beta_{g}\;\left(P_{I}-P_{g}\right)+\frac{D_{s}\beta_{s}A}{\frac{1}{2}L_{s}}\left(P_{s}-P_{g}\right)$$

**TABLE 1:** Model parameters and uncertainty ranges.

Symbol	Model parameters	Average value	Uncertainty (%)
*β*_b_	Solubility in blood*	232	±10
*β*_e_	Solubility in epidermis*	232	±20
*β*_s_	Solubility in stratum corneum*	211	±25
D_e_	Molecular diffusivity–epidermis (cm^2^·s^−1^)	5.0 × 10^−6^	±25
D_s_	Molecular diffusivity–stratum corneum (cm^2^·s^−1^)	5.0 × 10^−10^	±50
*L*_e_	Thickness of epidermis (cm)	0.02	±25
*L*_s_	Thickness of stratum corneum (cm)	0.0015	±25
*L*_g_	Thickness of gas compartment (cm)	0.5	±30
*A*_c_	Capillary surface area (cm^2^)	7.5 × 10^−2^	±50
$\dot{Q}$	Blood flow (mL·s^−1^)	4.0 × 10^−4^	±30
$\dot{V}$	Convective gas flow (mL·s^−1^)	5.0 × 10^−5^	±50

* Units for solubility are mL ethanol·100 mL medium^−1^·Torr^−1^.

The system of four ODEs was solved numerically to determine the partial pressure of ethanol in the epidermis, stratum corneum layers, and gas compartment as a function of time given a time varying arterial partial pressure of ethanol (BAC profile). Time derivatives were solved using LSODE, a time‐integrating algorithm developed by Hindmarsh (1981). The equations were solved in terms of partial pressures. Additionally, the mathematical model was imported into JSim, a Java‐based system for solving differential equations (Butterworth et al., 2013; Interagency Modeling Analysis Group, 2024). The JSIM model code was archived on the Interagency Modeling and Analysis Group website where instructions on downloading and running the model code were provided (Interagency Modeling and Analysis Group, 2024).

For presentation of results, the partial pressures of ethanol in the gas compartment were converted into equivalent BAC (BAC_EQ_) at 37°C using the following relationship.

(5) $$\mathrm{BA}C_{\mathrm{EQ}}=\frac{\beta_{b}}{\beta_{g}\mathrm{RT}}P_{g}$$

where R is the universal gas constant (62,360 Torr·cm^3^·mol^−1^·K^−1^), and *T* is the temperature (K).

### 2.2 Parameters

The criteria used for parameter value selection was described elsewhere (Anderson & Hlastala, 2006). A brief description is provided here. The skin tissue model has dimensions of 1 cm x 1 cm x L, where *L* is the thickness of each compartment. The “solubility” of ethanol in the gas phase, *β*_g_, is 0.132 mL ethanol 100 mL gas^−1^ Torr^−1^. For the 11 parameters, the average values, uncertainty ranges, and associated units are listed in Table 1. The average values were determined from a review of the scientific literature and correspond to the average dimensions and physical characteristics of healthy skin tissue (Anderson & Hlastala, 2006). For most of the parameters, the uncertainty ranges have not been quantified. Thus, the uncertainty ranges in Table 1 reflect the number of measurements and quality of information known about each parameter value. Smaller uncertainty ranges were assigned when parameters values were determined from careful or repeated measurements. Likewise, the probability distribution functions for these parameters are unknown. Thus, the simplest probability distribution function, a uniform (i.e., rectangular) probability distribution, was assumed for each parameter.

### 2.3 Simulations

The model simulated the movement of ethanol between the blood and air via diffusion through the skin. BAC profiles were defined by prescribing the absorption time, maximum BAC, and the metabolic elimination rate. Linear absorption and elimination rates were assumed. Absorption times ranged from 0.25 to 2.0 h in quarter‐hour increments. Maximum BAC (BAC_max_) values ranged from 0.02 to 0.10 g/dL in 0.02 g/dL increments. A metabolic elimination rate of 0.018 g/dL/h was assumed for all simulations. For model parameters listed in Table 1, average values were used. Within the gas compartment, only fresh air (i.e., *P*_I_ = 0) ventilation was simulated.

**FIGURE 2:**
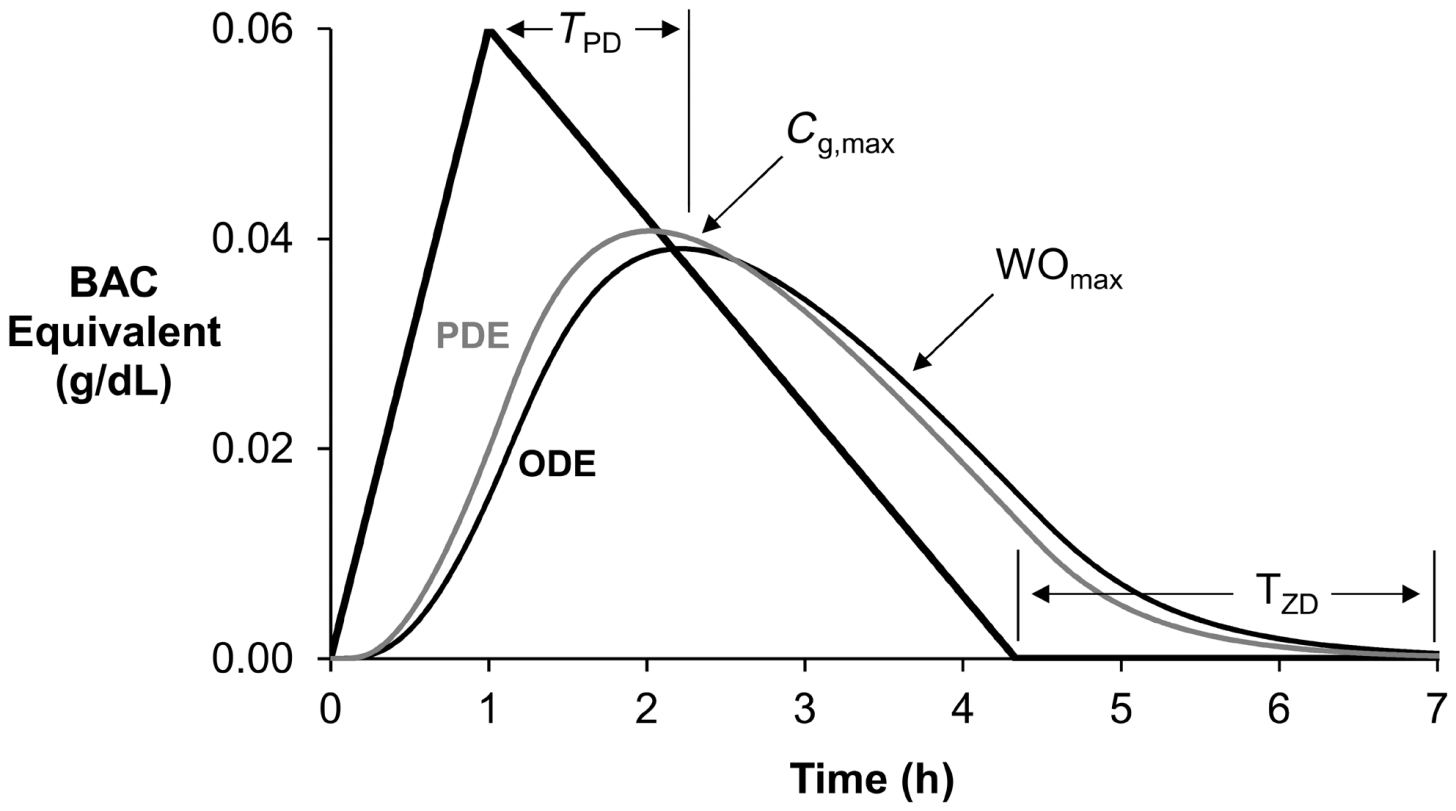
Transdermal ethanol curves using PDE and ODE models are shown with depiction of model outputs. BAC profile (thick black line) was imposed, and the equivalent ethanol concentration in the gas compartment (*C*_g_) was calculated using the ODE (thin black) and PDE (thin gray) models. The delay time between peaks (*T*_PD_) is the difference in time between the maximum BAC and maximum ethanol concentration in the gas compartment (*C*_g,max_). The maximum washout rate (WO_max_) was calculated as the negative slope of the *C*_g_ curve. *T*_ZD_ is the difference in time between zero ethanol concentration in the blood and gas space.

Model simulations resulted in gas phase ethanol profiles from which four values were calculated (Figure 2): (1) maximum equivalent ethanol concentration (*C*_g,max_, Equation 5), (2) maximum rate of decreasing ethanol concentration (WO_max_), (3) time difference (*T*_PD_) between BAC_max_ and *C*_g,max_, and (4) time difference between zero ethanol concentration (*T*_ZD_) in the blood and that in the gas space (defined as *C*_g_ = 0.001 g/dL). While all four of these outputs can be measured experimentally using current technology, in this study, these four outputs were compared to prior experimental measurements reported in the scientific literature. Additionally, the effect of absorption time and BAC_max_ on these four outputs was assessed and compared to the analogous relationships provided by the PDE model.

### 2.4 Sensitivity analysis

Nine sensitivity analyses using Latin hypercube sampling (Blower & Dowlatabadi, 1994) were performed to determine the sensitivity of the four model outputs described above and in Figure 2 to changes in the 11 model parameters listed in Table 1. For a single sensitivity analysis, the ODE model simulated the transdermal kinetics of ethanol 50 times with each simulation using a unique set of the 11 parameter values. The value of each parameter during each of the 50 simulations was randomly sampled. The rectangular probability distribution of each parameter was divided into 50 equal probability intervals. For each simulation, one interval for a given parameter was randomly sampled without replacement and the average value for that interval was assigned to be the parameter value. More detailed descriptions of this sampling method have been published (Anderson & Hlastala, 2006; Blower & Dowlatabadi, 1994; Bui et al., 1998).

The final step in the sensitivity analysis was to determine, for each output, a quantitative sensitivity index for each of the 11 parameters and establish a threshold to identify the significance of the sensitivity index. A partial rank correlation coefficient (PRCC) between each input variable (i.e., model parameter) and output variable was used for the quantitative sensitivity index (Blower & Dowlatabadi, 1994). To determine the significance of each sensitivity index, each PRCC value was tested using a two‐sided Student's *t*‐test to evaluate if it was statistically different from zero (*p* < 0.05), (Blower & Dowlatabadi, 1994). The sensitivity of the four outputs (*C*_g,max_, WO_max_, *T*_PD_, and *T*_ZD_) to changes in the 11 model parameters was determined.

To explore the effects of alcohol absorption and elimination on the sensitivity of model outputs to model parameters, nine sensitivity analyses were performed. For each of the nine analyses, a different BAC profile was specified by varying the absorption time of alcohol into the blood (0.5, 1, or 2 h) or the maximum BAC (0.02, 0.05, or 0.10 g/dL). The results were compared to the same analysis performed on the PDE model (Anderson & Hlastala, 2006).

## 3 RESULTS

For all simulations, solutions of the model equations were well behaved with no instances of negative results, mass imbalance, or dependence on changes in time step.

Supradermal alcohol measurements are surrogates, delayed in time and attenuated in magnitude, for measuring BAC. To allow easy comparison with BAC, SACs are converted into equivalent BACs for all presentations (Equation 5). Figure 2 compares the supradermal ethanol concentration, *C*_g_, calculated by both the ODE model and the prior PDE model to the imposed BAC values at corresponding time points. The imposed BAC profile has a rise time of 60 min, a maximum BAC (BAC_max_) of 0.06 g/dL, and a metabolic elimination rate of 0.018 g/dL/h. Using this BAC profile and the average model parameters (Table 1), the transport of ethanol through the skin is simulated using the ODE model. These results are compared to published results using the PDE model and the same model inputs (Anderson & Hlastala, 2006). The predicted *C*_g_ curves for the ODE (thin black) and the prior PDE (thin gray) models have very similar shapes and nearly overlap (Figure 2). As compared to the imposed BAC profile, both *C*_g_ curves show a maximum value, *C*_g,max_, that is attenuated (~2/3 of BAC_max_) and time‐delayed, *T*_PD_, by approximately 1 h. During the elimination phase, the *C*_g_ values decrease at slower rates (WO_max_) than the imposed BAC profile. Both models predict the *C*_g_ curve to be shifted right, attenuated, and spread relative to the BAC profile. These characteristics are a direct consequence of the stratum corneum diffusion barrier (see Section 3.1, Table 2, and Section 4).

The effects of different BAC profiles on the four model outputs (*C*_g,max_, WO_max_, *T*_PD_, and *T*_ZD_ defined in Figure 2) are examined. A variety of BAC profiles are specified by changing BAC_max_ and absorption time while maintaining a metabolic elimination rate of 0.018 g/dL/h for all BAC profiles. Simulations use average model parameter values (Table 1). Each model output for a given BAC absorption time is plotted against BAC_max_. Each curve corresponds to a BAC absorption time ranging between 0.25 and 2 h at 0.25 h intervals.

**FIGURE 3:**
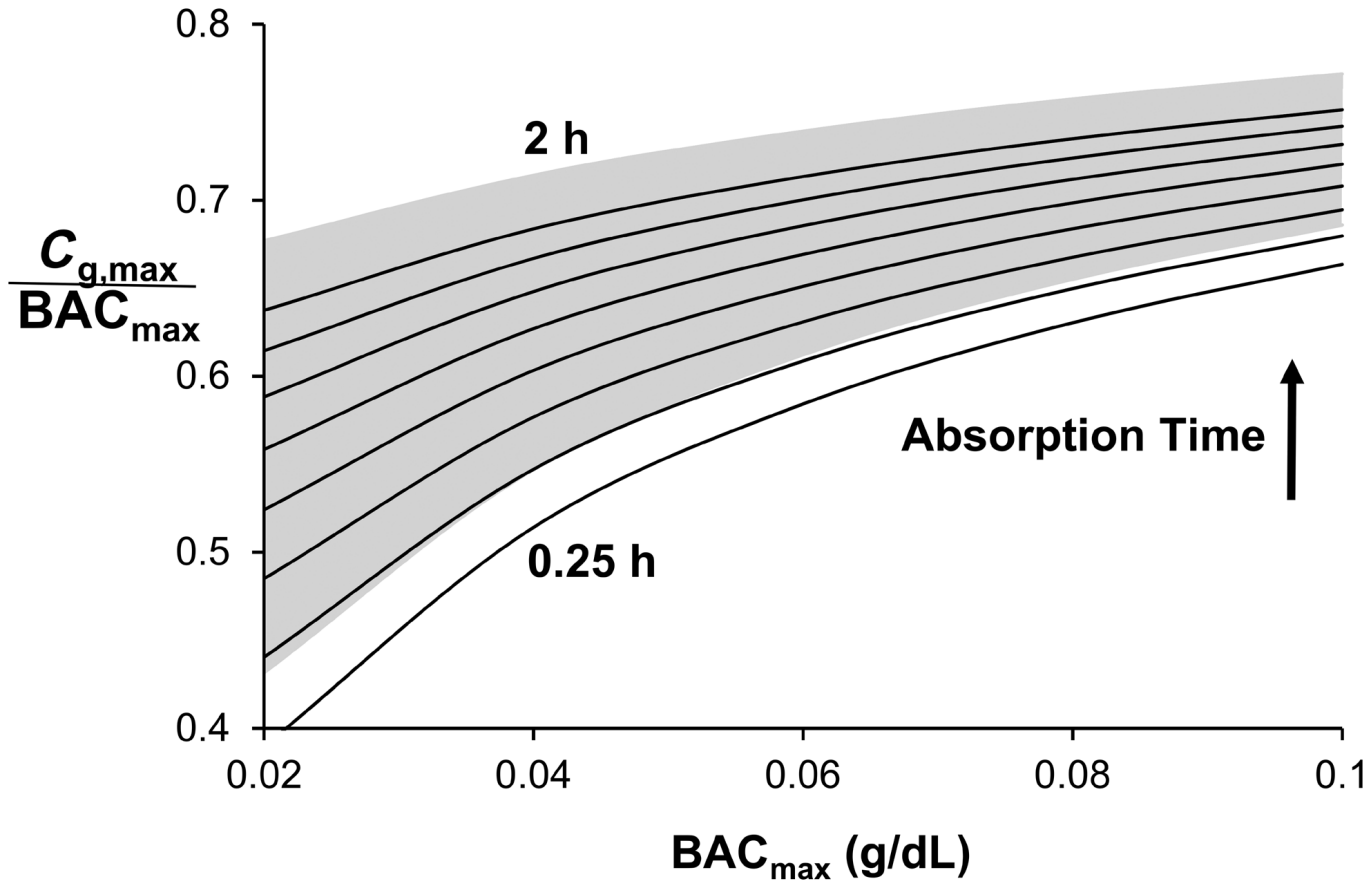
*C*_g,max_ normalized by BAC_max_ increases with BAC_max_ and absorption time. *C*_g,max_ never equals BAC_max_. Top and bottom curves represent 2‐ and 0.25‐h absorption times, respectively, with curves interposed at 0.25‐h increments. The gray area indicates the range of curves for the prior PDE model.

**FIGURE 4:**
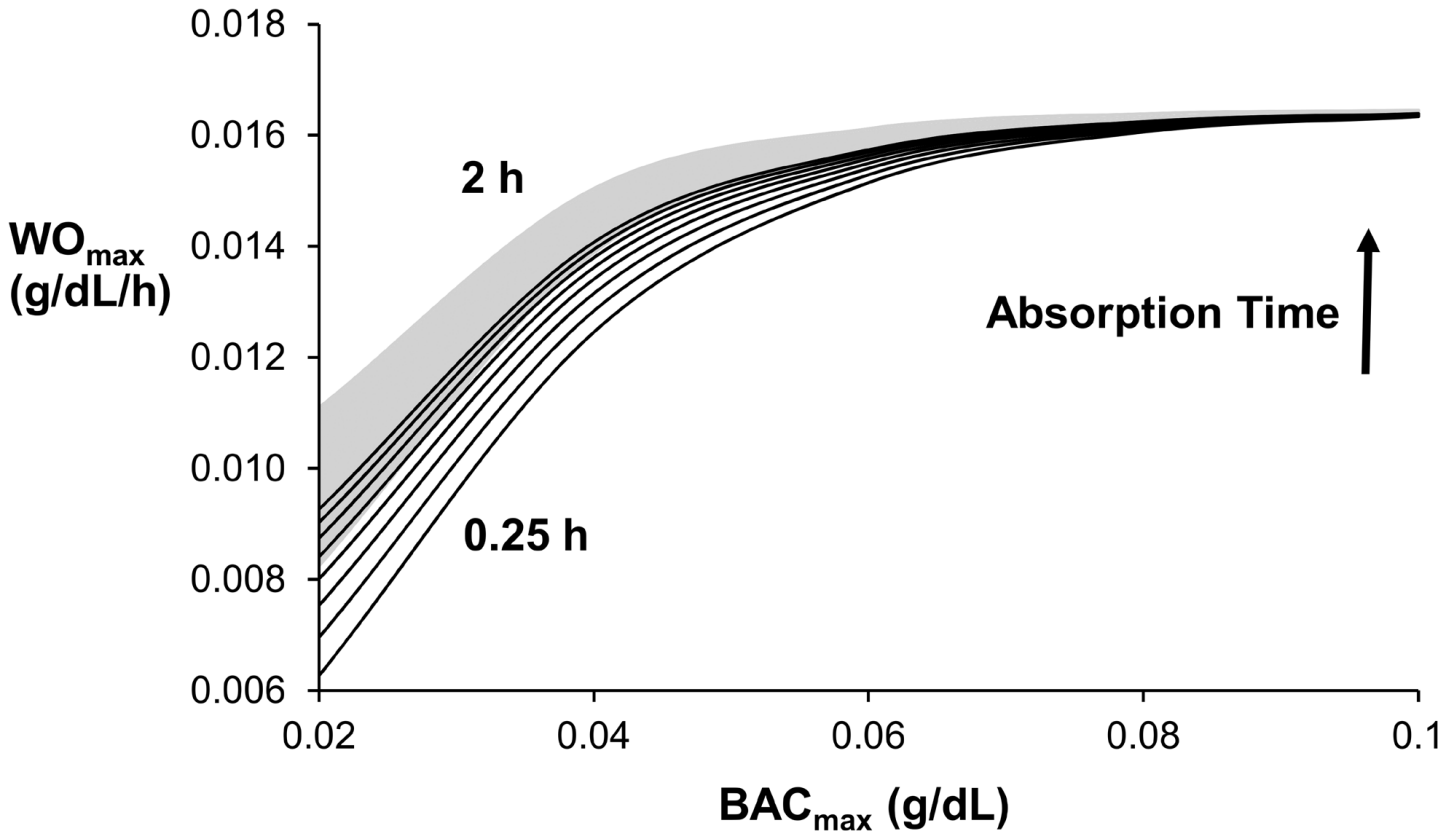
WO_max_ increases with increases in BAC_max_ and absorption time. When BAC_max_ ≥0.08 g/dL, WOmax = 0.0164 g/dL/h. Top and bottom curves represent 2‐ and 0.25‐h absorption times, respectively, with curves interposed at 0.25‐h increments. The gray area indicates the range of curves for the PDE model. The imposed metabolic elimination rate of ethanol in blood was 0.018 g/dL/h.

**FIGURE 5:**
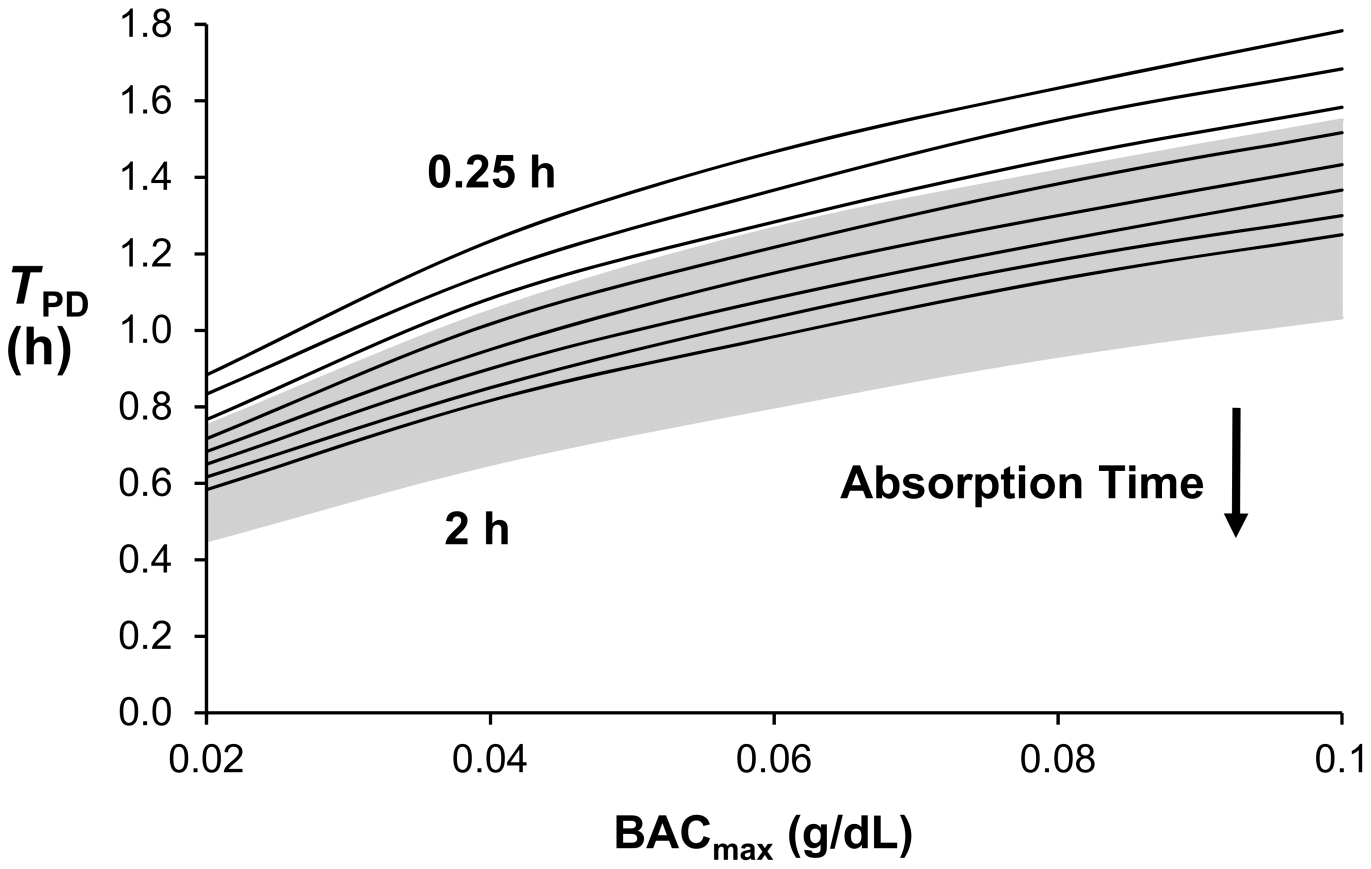
Time delay, *T*_PD_, between BAC_max_ and *C*_g,max_ increases as BAC_max_ increases or the absorption time decreases. A 30‐ to 100‐min delay exists between these two peaks for all cases shown. Top and bottom curves represent 0.25‐ and 2‐h absorption times, respectively, with curves interposed at 0.25‐h increments. The gray area indicates the range of curves for the PDE model.

**FIGURE 6:**
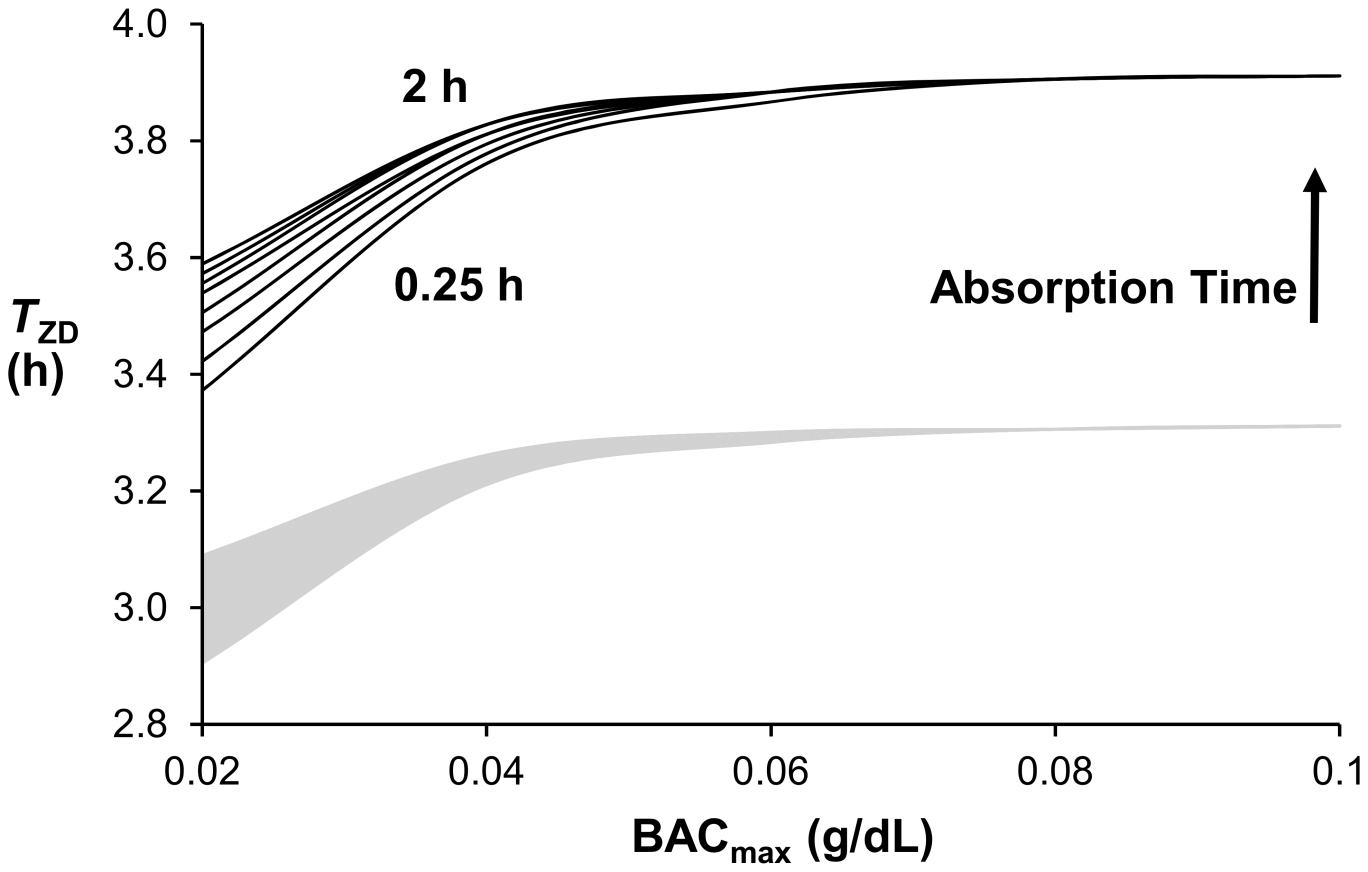
*T*_ZD_ increases modestly with increases in BAC_max_ and absorption time. When BAC_max_ ≥0.08 g/dL, *T*_ZD_ = 3.9 h. Top and bottom curves represent 2‐ and 0.25‐h absorption times, respectively, with curves interposed at 0.25‐h increments. The gray area indicates the range of curves for the PDE model.

The ODE and prior PDE models show the same relationship between a given model output and absorption time and a given model output and BAC_max_ (Figures 3, 4, 5, 6). Figure 3 shows that *C*_g.max_ is directly related to absorption time and BAC_max_. The ODE model predicts a smaller *C*_g,max_ than the prior PDE model for all conditions studied (solid lines as compared to gray area, respectively). Like *C*_g.max_, the maximum observed washout rate (WO_max_) is directly related to absorption time and BAC_max_ (Figure 4). When BAC_max_ <0.08 g/dL, the ODE model predicts a smaller WO_max_ than the prior PDE model. However, both models predict that WO_max_ asymptotes to ~ 0.0164 g/dL/h. when BAC_max_ ≥0.08 g/dL. Figure 5 shows T_PD_ to be directly related to BAC_max_ and inversely related to absorption time. The ODE model predicts a slightly longer time delay (~10 min) between peaks as compared to the prior PDE model. Figure 6 shows the time delay to zero, *T*_ZD_, between BAC and *C*_g_ to be directly related to absorption time and BAC_max_. Like the prior PDE model, *T*_ZD_ asymptotes when BAC_max_ ≥0.08 g/dL. However, the ODE model predicts a longer *T*_ZD_ (~30 min) for all conditions studied.

### 3.1 Sensitivity analysis

**FIGURE 7:**
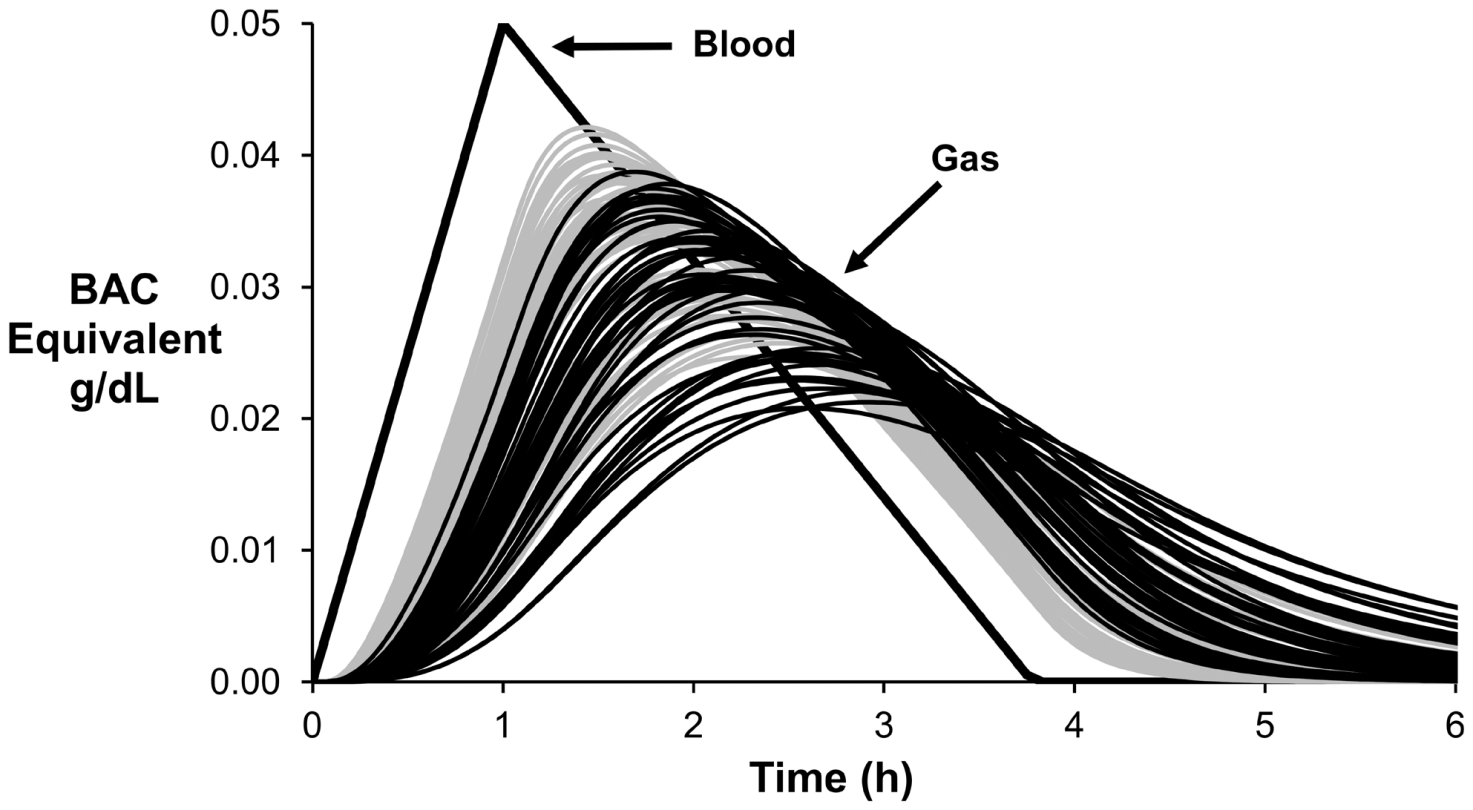
Large uncertainty in the 11 model parameters cause large variability in the supradermal alcohol curves for both the ODE (black curves) and PDE (gray curves) models. Fifty supradermal alcohol curves were predicted by both models using the 50 parameter sets selected by LHS. A single BAC profile (thick black curve) was imposed for all simulations: 1 h absorption time, BAC_max_ = 0.05 g/dL, and 0.018 g/dL/h. elimination rate.

Using LHS, nine sensitivity analyses were performed on the ODE model. The results were compared to the same analysis performed using the prior PDE model (Anderson & Hlastala, 2006). Figure 7 shows the *C*_g_ curves from 50 simulations using both the ODE model (black lines) and the prior PDE model (gray lines). The 50 *C*_g_ curves result from the 50 parameter sets randomly selected via LHS. In Figure 7, all simulations use a BAC profile having a 1‐h absorption time and BAC_max_ = 0.05 g/dL. Both models show large variation in *C*_g_ for the expected range of model parameters listed in Table 1. Consistent with Figure 2, the ODE model as compared to the prior PDE model shows, on average, a decreased maximum peak (*C*_g,max_), a greater time‐delay to the peak (*T*_PD_), a slower decrease in supradermal ethanol (WO_max_), and a greater time‐delay to return to zero (*T*_ZD_).

**TABLE 2:** Sensitivity analysis. C_g_ curves (Figure 7) from the ODE model using LHS were evaluated using PRCC.

Comp	Parameter	C_g,max_	WO_max_	Peak delay (T_PD_)	End delay (T_ZD_)
Gas	$\dot{V}$	−0.600*	−0.589*	−0.163	−0.320
	L_g_	−0.325	**−0.352**	0.420**	0.379*
Stratum corneum	*β*_s_	0.575**	0.568**	**−0.405**	**−0.344**
	D_s_	0.969**	0.967**	−0.941**	−0.956**
	*L*_s_	−0.950**	−0.948**	0.916**	0.952**
Epidermis	*β*_e_	0.114	0.123	0.054	0.042
	D_e_	**0.264**	0.321*	**−0.453**	−0.285
	*L*_e_	−0.305	**−0.339**	0.590**	0.359*
Blood	*β*_b_	0.040	−0.004	−0.100	−0.137
	$\dot{Q}$	−0.084	−0.083	0.090	0.085
	*A*_c_	0.406**	0.446**	−0.625**	**−0.356**

*Note*: The listed sensitivity coefficients (PRCC) are representative of the relationship between each model output (*N* = 4, Figure 2) and each model parameter (*N* = 11, Table 1). PRCC values are significant at the 0.05 level (bold), the 0.01 level (bold with *), or the 0.001 level (bold and **). Grayed boxes indicate statistically significant PRCC values reported previously for the PDE model (Anderson & Hlastala, 2006).

For the 50 LHS simulations presented in Figure 7, Table 2 summarizes the sensitivity relationship (using PRCC) between each model output (*N* = 4, Figure 2) and each model parameter (*N* = 11, Table 1). The sign in front of the PRCC value indicates the relationship between a parameter and an output. A negative (positive) PRCC value signifies an indirect (direct) relationship; that is, a decrease (increase) in the parameter will cause an increase (increase) in the output. The relationship (i.e., direct or indirect) between model outputs and model parameters was the same for both models irrespective of the statistical significance. While results for a single BAC profile are described, this sensitivity relationship and statistical significance between outputs and parameters is very similar for all nine BAC profiles studied.

For both models, all four outputs are statistically sensitive to the three parameters defining the stratum corneum: *D*_s_, *L*_s_, and *β*_s_. Of these, *D*_s_ and *L*_s_ have the greatest effect (i.e., greatest PRCC) on the model outputs over their uncertainty range. The parameters describing the gas compartment provide the next greatest effects on model outputs for both models. While the thickness of the gas compartment (i.e., volume) affects most all model outputs, the ventilation rate affects maximum gas concentration, *C*_g,max_, and the maximum rate of ethanol decrease, WO_max_. Unlike the prior PDE model, the ODE model has a significant sensitivity to the capillary surface area, *A*_c_, and two parameters that describe the epidermal compartment, *D*_e_ and *L*_e_.

## 4 DISCUSSION

Like the prior PDE model, ODE model predictions for *C*_g,max_, WO_max_, *T*_PD_, and *T*_ZD_ compare well to experimental measures from the scientific literature. The literature and ODE model show *C*_g,max_ to be less than BAC_max_ (Brown, 1985; Swift, 2000). When normalized by BAC_max_, the ratio of *C*_g,max_ to BAC_max_ (*C*_g,max_/BAC_max_) varied between 0.39 and 0.75 for the ODE model and between 0.29 and 0.50 from experimental measurements (Swift, 2000). Similarly, the maximum washout rate (WO_max_) was always less than the imposed metabolic elimination rate of 0.018 g/dL/h (Figure 4). Experimental measures demonstrate this finding (Brown, 1985). In both experimental measurements and model predictions, a time delay between peaks (*T*_PD_) exists and increases with BAC. For the ODE model, *T*_PD_ ranges from 30 to 110 min and increases with BAC (Figure 5). Experimental observations show: (1) *T*_PD_ ranges between 30 and 120 min (Brown, 1985; Lawson et al., 2019; Swift, 2000; Swift et al., 1992), and (2) *T*_PD_ increases from 30 min when BAC <0.1 g/dL to 120 min when BAC >0.15 g/dL (Swift et al., 1992). Finally, the time delay between when BAC = 0 and *C*_g_ = 0 (*T*_ZD_) is >2 h from experimental observation (Swift et al., 1992) and is approximately 4 h via the ODE model. These strong comparisons show the ODE model can closely predict the physiology and experimental measures of alcohol movement across the skin.

As compared to the PDE model, the ODE model predictions of *C*_g,max_/BAC_max_, WO_max_, and *T*_PD_ are very similar to those from the PDE model. On average, the ODE model shows a slightly greater attenuation of the peak alcohol concentration, a slightly smaller WO_max_, and a slightly greater *T*_PD_, when compared to similar measures from the PDE model. For the delay between zeros, *T*_ZD_ for the ODE model is, on average, 30 min greater than that for the PDE model. For *C*_g,max/_BAC_max_ and WO_max_, the greatest divergence occurs at small BAC_max_ whereas for *T*_PD_ and *T*_ZD_ the divergence is not dependent on BAC_max_ (Figures 3, 4, 5, 6).

In general, the ODE and PDE models perform very similarly. However, small differences in model predictions result from differences in model structure. The sensitivity analysis (Table 2) detailed these differences via changes in relationship between the model outputs and parameters. For both models, the model outputs were sensitive to parameters describing the gas phase and stratum corneum. For the epidermis and blood compartments, the PDE model outputs (*C*_g,max_ and WO_max_) were only sensitive to the ethanol solubility in the epidermis. However, the ODE model outputs were sensitive to diffusivity and thickness of the epidermis, as well as, the surface area of the blood compartment. It should be noted that a sizable portion of the sensitivity is likely caused by the large uncertainties associated with these parameters. Reducing the uncertainty of these parameters through in vivo tracer techniques or ex‐vivo experiments should improve model predictions (Anderson & Bassingthwaighte, 2007; Carlson et al., 2008; Young & Wagner, 1979).

To better understand the cause of the difference in SAC profiles between the ODE and PDE models, the six parameters describing the dermis and stratum corneum were, one‐at‐a‐time, varied by ±10% using the same BAC profiles as used in the ODE sensitivity analysis. Decreasing *L*_s_ by 10% caused the SAC profile from the ODE model to best match (using residual sum of errors) that from the PDE model. Because *L*_s_ helps define the diffusive conductance and the ethanol capacitance (i.e., effective tissue volume for dissolving ethanol) within the stratum corneum (Equation 3), an additional simulation, where both *L*_s_ and D_s_ were decreased by 10%, allowed the effects of *L*_s_ on ethanol capacitance to be isolated. These simulations demonstrate that the ODE model under accounts for diffusive conductance within the stratum corneum, which causes nearly 50% of the difference between the SAC profiles from the ODE and PDE models. Likewise, an underprediction of ethanol capacitance within the stratum corneum by the ODE model accounts for nearly 50% of the difference between the SAC profiles from the two models.

This under accounting for diffusive conductance and ethanol capacitance within the stratum corneum is a result of the different model structures, compartmental versus differential modeling, and the associated boundary conditions. The compartmental ODE model lumps the entire stratum corneum into a single description while the discretization associated with the PDE accounts for differential changes throughout the space and may explain the difference in ethanol capacitance. Likewise, different boundary conditions may explain the differential impact of diffusive conductance. The PDE model has a local flux condition that depends on the concentration gradient at the local boundary between the compartments; whereas, the ODE model uses a mass transfer coefficient that smooths the flux condition across each half of the adjacent compartments (i.e., not restricted to the local boundary). The local flux condition with discrete spatial resolution (PDE) versus the compartmental flux condition with lumped resolution likely drives the difference in diffusive conductance.

Because the models perform similarly, the advantages of the new approach, for most applications, outweigh its limitations. Whereas both models are described by four (Wang et al., 2019) differential equations, numerical integration of the ODE model only requires solution of four equations while numerical integration of the PDE model requires solution of 100s of equations to account for the spatial gradient of alcohol concentration (Anderson & Hlastala, 2006). The almost two‐orders of magnitude fewer equations means the ODE model can be solved more rapidly. The simplicity and increased solution speed of the ODE model will improve the utility of predicting BAC from measurements of SAC near the time of the drinking event. When alcohol is consumed, SAC rises from zero to a peak and then returns to zero at a rate consistent with alcohol elimination in the liver. SAC can be measured using commercially available devices (Wang et al., 2019). To date, transforming the SAC profile into a corresponding BAC profile is not performed by the measurement device because this “inverse” problem is computationally and, thus, energetically demanding. These wearable devices are designed for low energy consumption to maximize battery life. By decreasing the number of calculations required, timely predictions of BAC from SAC measurements are a possibility on a low‐power mobile device. Near “real‐time” predictions of BAC could improve the quality of information and timely decision making (e.g., elect to get a confirmatory test). In addition to solution speed, the system of ODEs can be better integrated into full body models of alcohol pharmacokinetics. Because these kinetic models typically use ODEs (Webster & Gabler, 2007, 2008), using a single type of equation throughout the model will produce a uniform model structure and decrease computational time. While the simpler ODE model improves computation speed and integration, the ODE model is limited in its ability to describe the spatial profile of alcohol throughout each tissue compartment.

When using mathematic models to interpret SAC or predict BAC from SAC, the effects of experimental conditions on skin transport properties and alcohol measurements must be minimized. For example, poorly understood water content of the skin can affect the movement of alcohol across the skin. The water content of the stratum corneum can vary due to dehydration, changes in atmospheric water content, and topical moisturizer (Blank et al., 1984; Wu et al., 1983). Increased water content can increase the thickness of stratum corneum, the solubility of ethanol in the stratum corneum, and the ethanol diffusion coefficient through stratum corneum (Dancik et al., 2013; Gajjar & Kasting, 2014; Scheuplein & Blank, 1971, 1973). Each of these factors can significantly affect the movement of ethanol across the skin (Table 2) and will give rise to the SAC variability shown in Figure 7. In addition to the effects on alcohol transport, the amount of water in the supradermal sample can affect the measurement of alcohol when using a fuel cell sensor, the most common sensor used in transdermal devices. Studies have shown that sweating affects skin alcohol measurement (Marques & McKnight, 2007; Wang et al., 2019). Additionally, changes in relative humidity are responsible for rapid fluctuations (i.e., “spikes”) in SAC (Li et al., 2021; Marques & McKnight, 2007). The common denominator is elevated water content, which is also known to affect the performance of fuel cells used to measure ethanol (Jalal et al., 2017, 2020). Because of this limitation, supradermal humidity should be measured and, in fact, some devices have included a sensor to measure water content and track relative humidity to improve measurement accuracy (Li et al., 2021; Wang et al., 2019).

Like the PDE model, the ODE model can be used to deconvolute (i.e., transform) SAC measurements into corresponding BAC. Instead of using BAC to calibrate the SAC deconvolution model, most studies have used BrAC measurements to calibrate the deconvolution model for SAC (Dai et al., 2016; Dumett et al., 2008; Luczak & Rosen, 2014; Rosen et al., 2014). However, investigators should use caution when calibrating against BrAC. Just like SAC is not BAC, breath alcohol is not blood alcohol. BrAC follows the arterial blood alcohol concentration whereas BAC measurements are venous in nature. As a result, BrAC can be much greater than BAC during the absorptive or rising phase (Hlastala & Anderson, 2016). More importantly, BrAC is significantly affected by a variety of breath factors including inhaled air volume, exhaled air volume, vital capacity, breathing pattern, and breath temperature (Anderson & Hlastala, 2019; Hlastala & Anderson, 2007, 2016). Without well‐controlled breath alcohol measurements, factors affecting breath alcohol concentration, like sex, height, and age which affect vital capacity, may confound the calibration of the SAC deconvolution model. For example, studies have shown that the sex of the subject can affect relationship between BrAC and SAC (Hill‐Kapturczak et al., 2015; Marques & McKnight, 2009). Because breath samples were used, it cannot be known if this relationship was driven by sex‐related factors affecting BrAC (as discussed above) or sex‐related factors affecting the skin (e.g., thickness and hydration) (Giacomoni et al., 2009; Luebberding et al., 2013). Thus, if SAC is compared and calibrated to BAC, unnecessary confounding by uncontrolled factors affecting breath alcohol measurement will be minimized.

## 5 CONCLUSION

This manuscript describes a new approach for modeling transdermal ethanol kinetics. The new ODE model details alcohol movement from the capillary blood, across two skin layers and into the gas above the skin using four ODEs. The key to implementing this approach was defining a mass transfer coefficient to describe diffusion between skin compartments. Outputs from the ODE model compared well with data from the literature. When compared to a prior model (PDE model) that used a mixture of PDEs and ODEs, the ODE model performed similarly. However, the ODE model as compared to the PDE model had slightly slower washout rates and slightly faster times to peak and zero SACs. Like the PDE model, a sensitivity analysis demonstrated the ODE model was sensitive, primarily, to changes in solubility and diffusivity within the stratum corneum, stratum corneum thickness, and the volume of gas above the skin. The small difference in SAC profiles between the ODE and PDE models was almost completely caused by differences in the mathematical description of diffusive conductance and ethanol capacitance. The simpler ODE model will streamline integration into larger physiologic models, reduce computation time to generate solutions, and decrease the time needed to transform SAC measurements to predicted BAC values.

## CONFLICT OF INTEREST STATEMENT

The author serves as an expert witness on alcohol related matters.

## Data Availability

The JSIM model code is archived on the Interagency Modeling and Analysis Group website and can be accessed via this web address: https://www.imagwiki.nibib.nih.gov/physiome/jsim/models/webmodel/NSR/transdermal‐ethanol‐transport‐ethanol‐diffusion‐through‐skin.
